# Do negative social ties accelerate aging in adults, or does aging erode social ties?

**DOI:** 10.1073/pnas.2608036123

**Published:** 2026-05-11

**Authors:** Quan Zhang

**Affiliations:** ^a^Department of Quantitative Health Sciences, John A. Burns School of Medicine, University of Hawaii, Honolulu, HI 96813; ^b^Department of Economics, College of Social Sciences, University of Hawaii, Honolulu, HI 96822; ^c^University of Hawaii Economic Research Organization, Honolulu, HI 96822

Lee et al. (1) report that people with more “hasslers” in their social network show signs of accelerated biological aging, measured through DunedinPACE and GrimAge2 epigenetic clocks. The headline result—roughly 9 mo of excess biological age per additional hassler—is striking. But the cross-sectional design makes it very hard to know what is driving what, and this deserves closer scrutiny.

The most pressing concern is reverse causality. Someone whose health is declining—from fatigue, chronic pain, or cognitive slowing—may become harder to get along with, or may perceive ordinary interactions more negatively. Lee et al. address this with a follow-up analysis showing that baseline hasslers predict subsequent self-reported health decline after adjusting for baseline health. However, this test uses subjective health ratings as outcomes, not the epigenetic clocks (DunedinPACE, GrimAge2) on which the paper’s central claims rest, and the short follow-up window cannot capture the slow feedback loops through which declining biological function reshapes social relationships over years. A Mendelian randomization study in the UK Biobank underscores the concern: Observational associations between social disconnection and disease were largely noncausal once genetic confounding was accounted for ref. 2.

Omitted confounders compound the problem. Cross-sectional network data cannot distinguish social influence from homophily or environmental confounding without longitudinal or experimental variation (3). Neuroticism, for instance, predicts both interpersonal conflict and elevated allostatic load across multiple biomarker systems, as shown in a recent meta-analysis of over 18,000 adults (4). The hassler classification is based on the respondent’s own perception. A neurotic or depressed person might label the same interaction as hostile that someone else would shrug off. This is not classical measurement error—it is systematically correlated with the outcome, biasing estimates away from zero even if the true effect were small.

The sample’s generalizability warrants scrutiny. All 2,685 participants come from a single Indiana cohort whose demographic and healthcare profile differs from the national population. Replication in broader samples—the Health and Retirement Study or longitudinal aging panels outside the United States—would clarify whether these associations hold up.

What would stronger evidence look like? Longitudinal data with individual fixed effects would control for stable traits like personality. Better still would be quasi-experimental approaches exploiting exogenous shocks to network composition—a spouse’s death, a forced relocation—as sources of variation in negative tie exposure. As Bramouillé et al. (5) discuss, separating genuine peer effects from correlated unobservables remains a central challenge in network research, and cross-sectional ego-centric data are ill-suited for it.

Finally, DunedinPACE itself (6), while a useful tool, has known sensitivity to batch effects and cell-type heterogeneity, issues that a recent review in *Nature Reviews Genetics* identifies as ongoing challenges for all epigenetic clocks (7). Reporting E-values (8) would give readers a concrete sense of how strong an unmeasured confounder would need to be to fully explain the observed associations. The relationship between social environment and biological aging is important, but getting the identification right matters—especially if these findings are meant to guide intervention.
